# Lentiviral Gene Therapy for Mucopolysaccharidosis II with Tagged Iduronate 2-Sulfatase Prevents Life-Threatening Pathology in Peripheral Tissues But Fails to Correct Cartilage

**DOI:** 10.1089/hum.2023.177

**Published:** 2024-04-16

**Authors:** Fabio Catalano, Eva C. Vlaar, Zina Dammou, Drosos Katsavelis, Tessa F. Huizer, Giacomo Zundo, Marianne Hoogeveen-Westerveld, Esmeralda Oussoren, Hannerieke J.M.P. van den Hout, Gerben Schaaf, Karin Pike-Overzet, Frank J.T. Staal, Ans T. van der Ploeg, W.W.M. Pim Pijnappel

**Affiliations:** ^1^ Department of Clinical Genetics, Erasmus MC University Medical Center, Rotterdam, The Netherlands.; ^2^ Department of Pediatrics, Erasmus MC University Medical Center, Rotterdam, The Netherlands.; ^3^ Center for Lysosomal and Metabolic Diseases, Erasmus MC University Medical Center, Rotterdam, The Netherlands.; ^4^ Department of Immunology, Leiden University Medical Center, Leiden, The Netherlands.; ^5^ Department of Pediatrics, Leiden University Medical Center, Leiden, The Netherlands.

**Keywords:** Hunter syndrome, Mucopolysaccharidosis type II, lentiviral gene therapy, IDS, IGF2, ApoE2, RAP, macrophage engraftment

## Abstract

Deficiency of iduronate 2-sulfatase (IDS) causes Mucopolysaccharidosis type II (MPS II), a lysosomal storage disorder characterized by systemic accumulation of glycosaminoglycans (GAGs), leading to a devastating cognitive decline and life-threatening respiratory and cardiac complications. We previously found that hematopoietic stem and progenitor cell-mediated lentiviral gene therapy (HSPC-LVGT) employing tagged IDS with insulin-like growth factor 2 (IGF2) or ApoE2, but not receptor-associated protein minimal peptide (RAP12x2), efficiently prevented brain pathology in a murine model of MPS II. In this study, we report on the effects of HSPC-LVGT on peripheral pathology and we analyzed IDS biodistribution. We found that HSPC-LVGT with all vectors completely corrected GAG accumulation and lysosomal pathology in liver, spleen, kidney, tracheal mucosa, and heart valves. Full correction of tunica media of the great heart vessels was achieved only with *IDS.IGF2co* gene therapy, while the other vectors provided near complete (*IDS.ApoE2co*) or no (*IDSco* and *IDS.RAP12x2co*) correction. In contrast, tracheal, epiphyseal, and articular cartilage remained largely uncorrected by all vectors tested. These efficacies were closely matched by IDS protein levels following HSPC-LVGT. Our results demonstrate the capability of HSPC-LVGT to correct pathology in tissues of high clinical relevance, including those of the heart and respiratory system, while challenges remain for the correction of cartilage pathology.

## INTRODUCTION

Mucopolysaccharidosis type II (MPS II) (Hunter syndrome, OMIM 309900) is an X-linked lysosomal storage disorder (LSD) characterized by a generalized accumulation of partially digested heparan sulfate and dermatan sulfate, with manifestations affecting multiple tissues and organs. Two-third to three-fourth of patients have the neuronopathic form of the disease, leading to severe and progressive neurological decline. Other manifestations affect both neuronopathic and non-neuronopathic patients and involve upper respiratory issues, valvular heart disease, hepatosplenomegaly, joint stiffness, and bone and cartilage abnormalities, leading to poor growth.^1–11^

Intravenously administered enzyme replacement therapy (ERT)—the standard treatment for MPS II—does not reach the brain due to the blood–brain barrier. In addition, other aspects of MPS II pathology are also poorly targeted by ERT and, among these, respiratory and cardiac complications represent the main cause of death, while pathology in cartilage strongly impacts growth and mobility.^2–5,10–14^ It is therefore imperative that emerging treatments for MPS II—including the ones designed to target the brain—are also tested for their efficacy to prevent or delay pathology in peripheral tissues.

We previously found^15^ correction of brain pathology of *Ids^y/−^* mice after hematopoietic stem and progenitor cell-mediated lentiviral gene therapy (HSPC-LVGT) with vectors encoding iduronate 2-sulfatase (IDS) tagged with either insulin-like growth factor 2 (IGF2)^16,17^ or receptor-associated protein minimal peptide (RAP12x2),^18^ as well as with the previously published fusion protein IDS.ApoE2,^19,20^ driven by the ubiquitous MND promoter. We showed that tagging of IDS with IGF2 and ApoE2, but not RAP12x2, improves prevention of brain pathology of *Ids^y/−^* mice. In this study, we show that HSPC-LVGT using tagged IDS prevents peripheral pathology in tissues considered critical for survival of patients, and we provide insight into the mechanism of action, as well as its limitations in reaching tissues such as cartilage.

## MATERIALS AND METHODS

Materials and Methods section can be found in the Supplementary Data.^45–49^

## RESULTS

### Gene therapy results in systemic delivery of IDS protein

HSPCs derived from CD45.1 *Ids*^y/−^ donor mice were transduced with either *IDSco*, *IDS.IGF2co*, *IDS.ApoE2co*, or *IDS.RAP12x2co* lentiviral vectors and transplanted in lethally irradiated 8–10 weeks old CD45.2 *Ids*^y/−^ mice^21,22^ (Table 1).^15^ Six months after gene therapy, average bone marrow vector copy numbers were between 2 and 3 copies per genome, while bone marrow chimerism values were 80–90% for all the treatment groups. This caused supraphysiological IDS enzyme activity in bone marrow and plasma at levels 30- to 150-fold above levels in wild-type (WT) mice (Table 1).

**Table 1.** VCN, chimerism and IDS activity after lentiviral gene therapy

Transgene (C-Terminal Tagging)	Linker	Tag	VCN Per Diploid Genome in Bone Marrow (PSI/Albumin)	Chimerism in Bone Marrow (CD45.1^+^ CD45.2^−^)	IDS Activity in Bone Marrow (nmol/mg × 4 h)	IDS Activity in Plasma (nmol/mg × 4 h)	n
IDSco	N/A	N/A	2.351 ± 1.329	91.103 ± 2.577	5,215.7 ± 1,160.4	130.09 ± 34.51	6
IDS.IGF2co	(LGGGGS)x4	AA 1, 8–67 hIGF2	2.953 ± 1.132	81.167 ± 18.104	4,344.4 ± 1,353.8	66.83 ± 15.66	6
IDS.ApoE2co	(LGGGGS)x4	AA (141–149)x2 hApoE	2.595 ± 1.694	84.585 ± 7.347	3,504.4 ± 1,952.4	133.76 ± 54.62	10
IDS.RAP12x2co	(LGGGGS)x4	AA (151–262) Gly (151–262) hRAP	1.982 ± 1.613	81.954 ± 19.196	2,893.2 ± 2,187.4	107.68 ± 53.46	7
GFP	N/A	N/A	0.610 ± 0.261	N/A	9.5 ± 8.5	0.31 ± 0.02	6
*Ids y/−* untreated	N/A	N/A	0.004 ± 0.001	4.4975 ± 3.282	11.6 ± 4.9	0.51 ± 0.37	6
WT untreated	N/A	N/A	0.003 ± 0.000	6.0701 ± 2.678	40.5 ± 32.8	1.92 ± 0.22	6

Gene therapy-treated mice were irradiated with an irradiation dose of 9 Gray and transplanted with 10^6^ hematopoietic stem and progenitor cells transduced at a multiplicity of infection of 1.

Chimerism for the GFP group could not be measured due to the overlap of the GFP and FITC excitation spectra (see Materials and Methods).

AA, amino acid; ApoE, Apolipoprotein E; co, codon optimized; GFP, green fluorescent protein; h, human; IDS, iduronate 2-sulfatase; IGF2, insulin-like growth factor; LGGGGS, lysine–glycine–glycine–glycine–glycine–serine; N/A, not applicable; RAP, receptor-associated protein; PSI, lentiviral packaging signal; VCN, vector copy number per diploid genome; WT, wild type.

**Figure 1.**
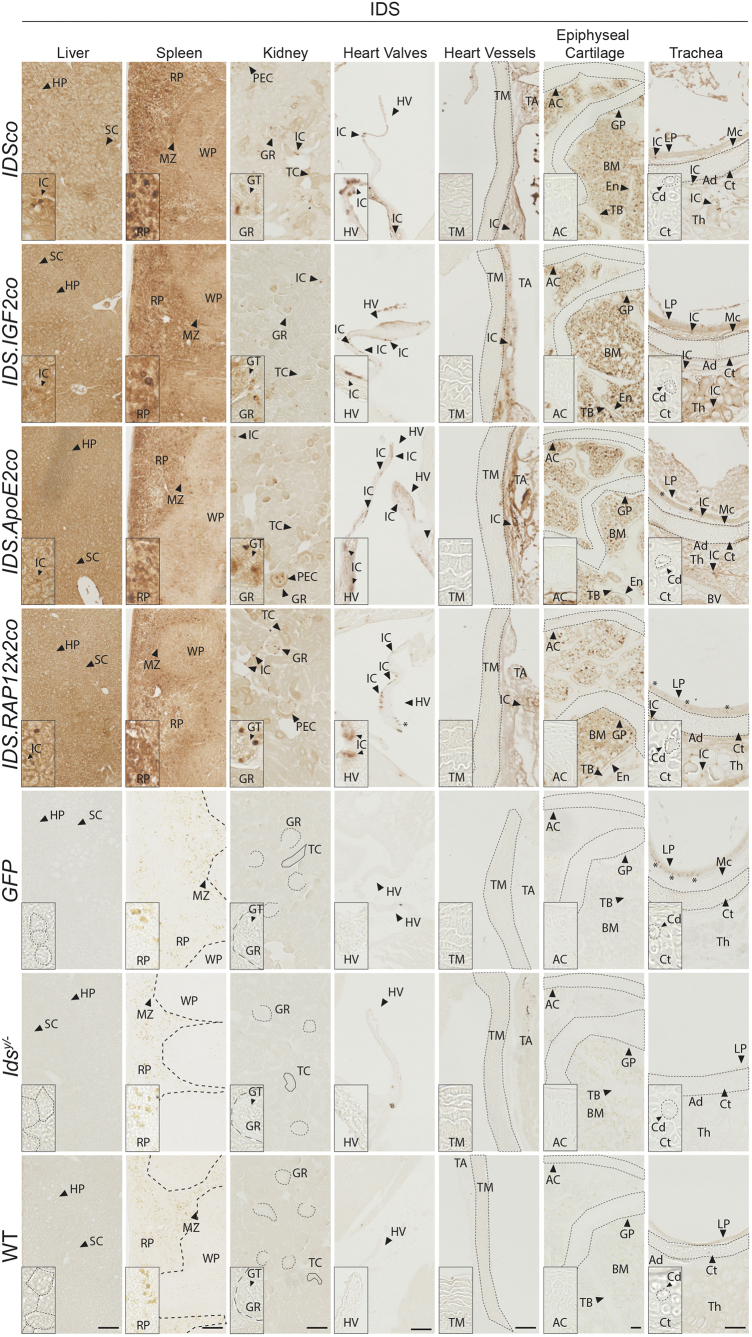
Biodistribution of IDS protein after gene therapy. Two-month-old *Ids^y/−^* mice were treated with gene therapy using 9 *Gray* (Gy) total body irradiation and multiplicity of infection of 1 (Table 1). Representative pictures of IDS staining of liver, spleen, kidney, epiphyseal cartilage, trachea, heart valves, and great heart vessels of gene therapy-treated *Ids^y/−^* mice and controls. In kidney, *dashed lines* indicate the Bowman's capsule of the glomerulus, while *solid lines* indicate tubules. *Dashed lines* in liver represent hepatocytes. Enlarged pictures for the articular cartilage of the knee and for trachea are shown in Supplementary Fig. S1 (knee joint) and Supplementary Fig. S2 (trachea). Inserts represent regions of interest magnified four times. *Indicate artefacts. *n* = *3* biological replicas. *Scale bar* = 100 μm. AC, articular cartilage; Ad, tracheal adventitia; BM, bone marrow; Cd, chondrocytes; Ct, hyaline cartilage of the trachea; En, endosteum; GP, growth plate; GR, glomerulus; GT, glomerular tuft; HP, hepatocytes; HV, heart valve; IC, interstitial cells; IDS, iduronate 2-sulfatase; LP, lamina propria of the tracheal mucosa; Mc, tracheal mucosa; MZ, marginal zone; PEC, parietal epithelial cells of the Bowman's capsule; RP, spleen red pulp; SC, sinusoids; TA, tunica adventitia; TB, trabecular bone; TC, tubules; Th, thyroid gland; TM, tunica media; WP, spleen white pulp; WT, wild type.

Gene therapy resulted in delivery of IDS protein to peripheral tissues at levels that were similar for all vectors tested (Fig. 1; Supplementary Figs. S1, S2, and S9). IDS immunoreactivity varied depending on the tissue analyzed and was either diffuse across the tissue section (liver; spleen; bone marrow; epiphyseal cartilage) or present in isolated cells (hereafter referred to as interstitial cells [IC]), while *GFP*-treated and untreated *Ids^y/−^* and WT mice did not show any IDS immunoreactivity in the tissues analyzed (Fig. 1).

**Figure 2.**
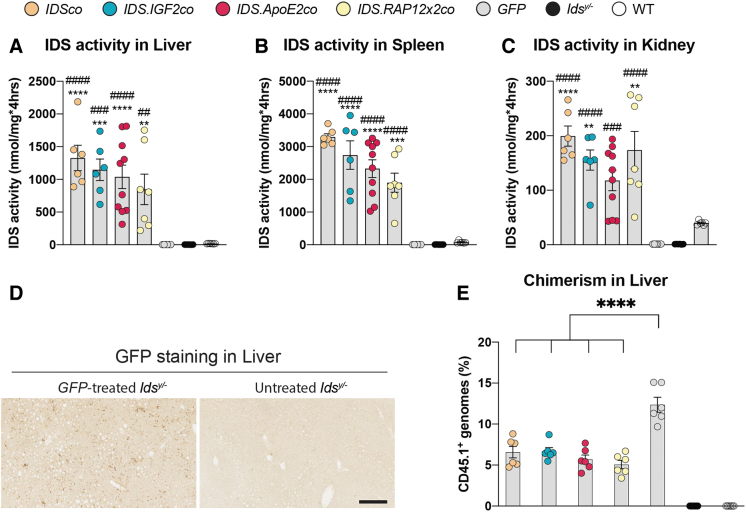
IDS activity in liver, spleen, and kidney after gene therapy. IDS enzyme activity in liver **(A)**, spleen **(B),** and kidney **(C)**. **(D)** Sections of liver of *GFP*-treated and untreated *Ids^y/−^* mice stained with anti-GFP antibody. **(E)** Chimerism analysis of liver measured by allele-specific qPCR on the *Cd45.1* locus. Data represent means ± SEM and were analyzed by one-way ANOVA followed by Bonferroni's multiple testing correction. Enzyme activity: *IDSco*, *IDS.IGF2co*, *GFP*, *Ids^y/−^*, and WT *n* = *6*; *IDS.ApoE2co n* = *10*; *IDS.RAP12x2co n* = *7*. Chimerism: *n* = *6.* GFP staining: *n* = *3.* **p ≤ 0.01, ***p ≤ 0.001, ****p ≤ 0.0001. ^##^*p* ≤ 0.01, ^###^*p* ≤ 0.001, ^####^*p* ≤ 0.0001. *Asterisks* represent significance versus WT; *hash* represent significance versus *Ids^y/−^*. Significant results are indicated by *brackets*. ANOVA, analysis of variance; GFP, green fluorescent protein; qPCR, quantitative polymerase chain reaction; SEM, standard error of the mean.

In liver, hepatocytes and sinusoids (SC) stained for IDS protein at similar levels after gene therapy with IDS and irrespective of the presence of a tag, with some IC occasionally showing increased levels (Fig. 1, liver inserts). In spleen of gene therapy-treated mice, IDS staining was present in the white pulp at weaker levels compared with the pronounced signal detected in the hematopoietic tissue of the red pulp (RP; Fig. 1, spleen inserts). In agreement, liver and spleen homogenates of WT mice showed 6 (liver) and 11 (spleen)-fold higher IDS enzyme activity, respectively, compared with homogenates from *GFP*-treated and untreated *Ids^y/−^* mice (Fig. 2A, B). In these tissues, gene therapy caused supraphysiological levels of activity at values ∼30 (spleen) and ∼70 (liver) times higher than WT mice for all the vectors.

In kidney, IDS immunoreactivity was weaker compared with liver and spleen and was mainly restricted to cells of the glomerular tuft (GT) and to IC in between the tubules (Fig. 1, kidney inserts). In the rest of the kidney tissue, some tubules (TC) and parietal epithelial cells (PEC) of the Bowman's capsule showed an increased and diffuse IDS staining above the background (Fig. 1, kidney inserts). IDS enzyme activity assay of kidney homogenates of gene therapy-treated mice revealed values that were lower compared with liver and spleen (∼10 times lower), and only 4 times higher than WT mice (Fig. 2C).

In heart, immunoreactivity was detected in IC located in the heart muscle (Supplementary Fig. S3A), in IC located in the endothelium of the heart valves (HV) and in the tunica adventitia (TA) of the great heart vessels. In addition, we also observed a weak and diffuse IDS immunoreactivity in the heart muscle and TA of the great heart vessels, above the background levels (Fig. 1, HV and heart vessel inserts; Supplementary Fig. S3A). No IDS protein was detected in the internal layers of the valve leaflet and in the tunica media (TM) of the heart vessels (Fig. 1, HV and heart vessel inserts).

Articular cartilage (AC) and growth plate (GP) cartilage of the femur and the tibia (Fig. 1, epiphyseal cartilage inserts; Supplementary Fig. S1), as well as the hyaline cartilage of the trachea (Fig. 1, tracheal cartilage inserts; Supplementary Fig. S2) were completely devoid of IDS staining after gene therapy. In contrast, gene therapy resulted in a diffuse immunoreactivity in the bone marrow tissue surrounding the AC and the GP, with some bone marrow cells showing a higher staining compared with the neighboring marrow (Fig. 1, epiphyseal cartilage inserts; Supplementary Figs. S1 and S2). In bones, we also observed IDS immunoreactivity in cells embedded in the trabecular bone (TB) and cells of the endosteum. Gene therapy also resulted in IDS staining in the tissues surrounding the tracheal cartilage, with some of the IC located in the mucosa (Mc), adventitia (Ad), or dispersed in the thyroid gland (Th) showing higher levels of IDS immunoreactivity compared with the rest of the tissue (Fig. 1, trachea inserts).

**Figure 3.**
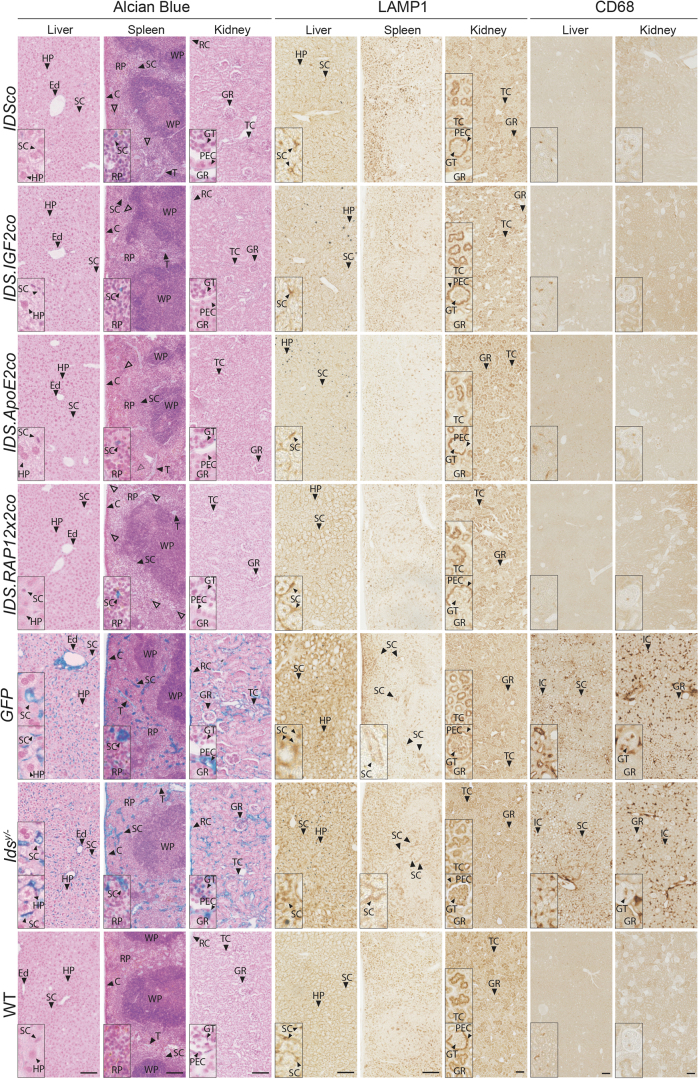
Gene therapy corrects pathology in liver, spleen, and kidney. Representative pictures of Alcian Blue, and LAMP1 and CD68 stainings of liver, spleen, and kidney of gene therapy-treated *Ids^y/−^* mice and controls. CD68 staining in spleen is shown in Supplementary Fig. S3. *Hollow arrowheads* indicate Alcian Blue-positive cells in gene therapy-treated mice. Inserts represent regions of interest magnified four times. *n* = *3* biological replicas. *Scale bars* = 100 μm. C, splenic capsule; Ed, endothelial cells; LAMP1, lysosomal-associated membrane protein 1; RC, renal capsule; T, splenic trabeculae.

We next investigated engraftment of donor-derived cells in the liver (Fig. 2D, E). In *GFP*-treated *Ids^y/−^* mice, we observed widespread GFP immunoreactivity throughout the whole tissue section, suggesting the presence of donor-derived cells (Fig. 2D). We confirmed that GFP-positive cells were donor derived by measuring chimerism in liver of *GFP*-treated mice, as well as in liver of mice treated with the other vectors (Fig. 2E). Gene therapy resulted in ∼6% chimerism in livers of mice treated with *IDSco*, *IDS.IGF2co*, *IDS.ApoE2co*, *IDS.RAP12x2co*, while *GFP*-treated mice showed ∼2-fold higher chimerism (∼12%) compared with the rest of the conditions (Fig. 2E), suggesting increased recruitment of donor-derived cells in livers of untreated and *GFP-*treated *Ids^y/−^* mice. This could be a consequence of an inflammatory state present in livers of *GFP* and *Ids^y/−^* mice compared with mice treated with the other vectors, as shown by the CD68 immunoreactivity levels detected in liver of *GFP* and *Ids^y/−^* mice. This suggests the presence of donor-derived infiltrating macrophages (Fig. 3).

In conclusion, gene therapy resulted in delivery of IDS protein to peripheral tissues at levels that were largely independent of the vector used and that depended on the tissue analyzed. Importantly, IDS protein could not be detected in cartilage tissues, as well as in the internal layers of the HV and in the TM of the great heart vessels.

### Alleviation of alcian blue, lysosomal-associated membrane protein 1 and CD68 by gene therapy

**Figure 4.**
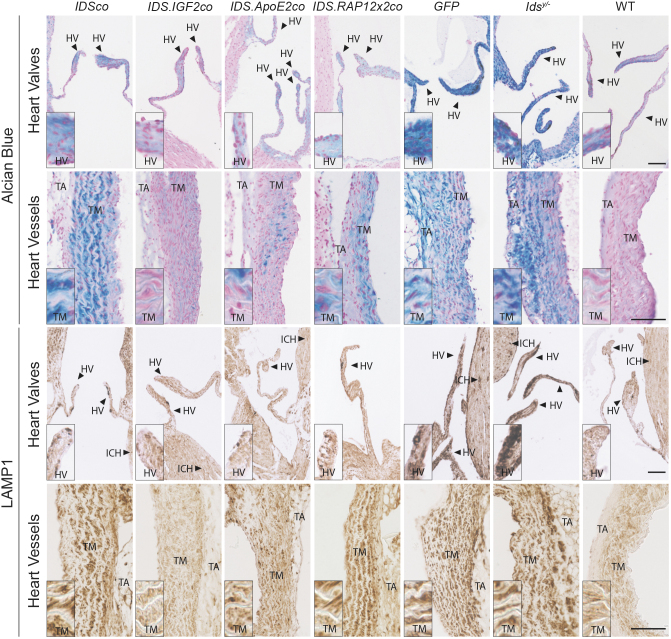
Gene therapy with *IDS.IGF2co* corrects pathology in heart. Alcian Blue and LAMP1 staining of heart valves and great heart vessels of *Ids^y/−^* mice after gene therapy and controls. Representative pictures are shown. Inserts represent regions of interest magnified four times. *n* = *3* biological replicas. *Scale bars* = 100 μm. ICH, interstitial cell of the heart muscle.

**Figure 5.**
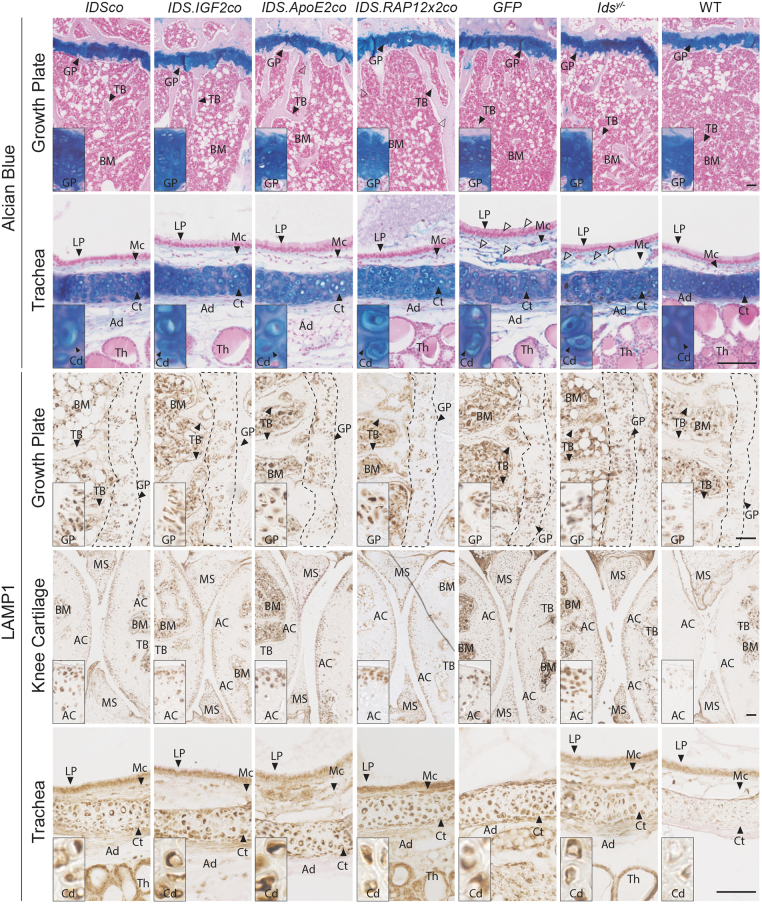
Gene therapy fails to correct cartilage pathology. Representative pictures of Alcian Blue and LAMP1 stainings of growth plate, articular cartilage, and trachea of *Ids^y/−^* mice after gene therapy and controls. *Enlarged pictures* for the articular cartilage of the knee and for trachea are shown in Supplementary Fig. S4 (Alcian Blue staining of knee joint), Supplementary Fig. S5 (Safranin O/Fast Green staining of the proximal epiphysis of the tibia), Supplementary Fig. S6 (Alcian Blue staining of trachea), Supplementary Fig. S7 (LAMP1 staining of the knee joint) and Supplementary Fig. S8 (LAMP1 staining of trachea). Inserts represent regions of interest magnified four times. *n* = *3* biological replicas. *Scale bars* = 100 μm. MS, menisci.

**Figure 6.**
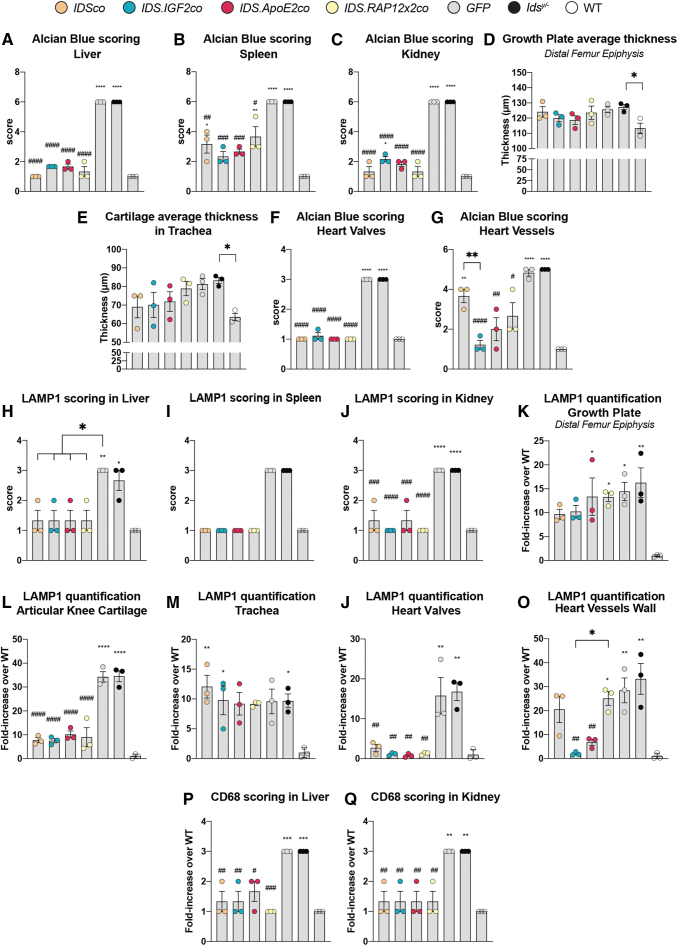
Quantification and scoring of Alcian Blue, LAMP1 and CD68 in peripheral organs. **(A–G)** Scoring of Alcian Blue in peripheral organs and analysis of cartilage thickness. Scoring of Alcian Blue in liver **(A)**, spleen **(B)**, kidney **(C)**, heart valves **(F)**, and heart vessels **(G)**. Scoring rules for Alcian Blue staining are shown in Supplementary Table S1 (liver, spleen, and kidney), Supplementary Table S2 (heart valves) and Supplementary Table S3 (great heart vessels). Cartilage thickness was measured in the growth plate of the distal epiphysis of the femur **(D)** and in trachea **(E)**. **(H–O)** Scoring and quantification of LAMP1 pathology. Scoring of LAMP1 pathology in liver **(H)**, spleen **(I)**, and kidney **(J)**. Scoring rules for LAMP1 staining are shown in Supplementary Table S4 (liver and spleen) and Supplementary Table S5 (kidney). Quantification of LAMP1 signal in the growth plate of the distal epiphysis of the femur **(K)**, articular knee cartilage **(L)**, trachea **(M)**, heart valves **(N)**, and in great heart vessels **(O)**. Scoring of CD68 pathology in liver **(P)** and kidney **(Q)** using the scoring rules shown in Supplementary Table S6 (liver and kidney). Data represent means ± SEM and were analyzed by one-way ANOVA followed by Bonferroni's multiple testing correction. *p ≤ 0.05, **p ≤ 0.01, ***p ≤ 0.001, ****p ≤ 0.0001. ^#^*p* ≤ 0.05, ^##^*p* ≤ 0.01, ^###^*p* ≤ 0.001, ^####^*p* ≤ 0.0001. *Asterisks* represent significance versus WT; *hash* represent significance versus *Ids^y/−^*. Significant results are indicated by *brackets*.

Below we present Alcian Blue, CD68 (Cluster of Differentiation 68) and lysosomal-associated membrane protein 1 (LAMP1) stainings in Figs. 3–5, while the respective quantifications are shown in Fig. 6. Alcian Blue is a polyvalent basic dye that binds to acidic polysaccharides in tissues.^23^ When employed at pH 1, like in the experiments presented in this study, it stains specifically for sulfated mucins such as heparan sulfate and dermatan sulfate accumulating in MPS II.^24^ CD68, a glycoprotein that is highly expressed by human monocytes and tissue macrophages, can indicate proliferation of macrophages responding to tissue damage or to inflammation.^25^ Finally, LAMP1 is a protein located in the lysosomal membrane, involved in lysosomal stability and pH sensing. LAMP1 can reflect the abundance or enlargement of lysosomes, which is characteristic of LSDs, including MPS II.^26^ Heparan and dermatan sulfate accumulation, as well as inflammation and lysosomal pathology are all hallmarks of MPS II pathology.

#### Liver, spleen, and kidney

In liver of *GFP*-treated and untreated *Ids^y/−^* mice, Alcian Blue staining was present in endothelial cells (Ed) of the hepatic vessels and hepatic SC (Fig. 3, Alcian Blue in liver), while in spleen, Alcian Blue staining was observed in SC and cells of the RP, splenic capsule (C), and trabeculae (T) (Fig. 3, Alcian Blue in spleen). Kidney showed Alcian Blue staining in the renal capsule, in TC, in the GT, and in the PEC of the Bowman's capsule (Fig. 3, Alcian Blue in kidney). *Ids^y/−^* mice and *GFP*-treated mice also showed increased LAMP1 immunoreactivity in sinusoidal structures of the liver and spleen (SC) compared with WT mice (Fig. 3, LAMP1 in liver and spleen). In kidney of *Ids^y/−^* mice, we observed increased LAMP1 staining in the GT and decreased staining in the parietal sheet (PEC), in addition to an increased number of TC showing LAMP1 immunoreactivity compared with WT mice (Fig. 3, LAMP1 in kidney).

*Ids^y/−^* mice and *GFP*-treated mice also showed an increased CD68 staining in liver and kidney (Fig. 3, CD68), while we did not observe obvious differences in the levels of CD68 staining between spleens of WT and *GFP*-treated and untreated *Ids^y^*^/−^ mice (Supplementary Fig. S3). CD68 immunoreactivity in liver was present in cells in proximity to hepatic SC or interstitial structures (IC) (Fig. 3, CD68 in liver), while in kidney CD68 staining was present in IC and in cells of the GT (Fig. 3, CD68 in kidney).

Gene therapy with all the vectors normalized Alcian Blue, LAMP1 and CD68-related pathology in liver and kidney (Fig. 3, liver and kidney inserts; Fig. 6A, H, P for liver; Fig. 6C, J, Q for kidney). In spleen, Alcian Blue staining was significantly reduced after gene therapy with all the vectors, while LAMP1 immunoreactivity was completely normalized (Fig. 3, spleen inserts; Fig. 6B, I).

#### HV and heart vessels

HV of *Ids^y/−^* and *GFP*-treated mice strongly stained with Alcian Blue (Fig. 4, Alcian Blue in HV). Additionally, in *Ids^y/−^* mice we observed the presence of a sparse Alcian Blue-positive staining in IC within the cardiac muscle, and in the TM and TA of the great heart vessels (Fig. 4, Alcian Blue in heart vessels), while no Alcian Blue staining was detected in muscle fibers (data not shown). Alcian Blue staining matched LAMP1 immunoreactivity as *Ids^y/−^* and *GFP*-treated mice also displayed high levels of LAMP1 immunoreactivity in the HV and in the TM of the great heart vessels, as well as an increased LAMP1 staining in IC of the cardiac muscle compared with WT animals (Fig. 4, LAMP1 in HV and heart vessels).

Gene therapy with all the vectors reduced Alcian Blue and LAMP1 staining in HV (Fig. 4, HV inserts; Fig. 6F, N), while in heart vessels, only gene therapy with *IDS.IGF2co* resulted in normalization, with *IDS.ApoE2co* gene therapy causing a near complete correction of Alcian Blue and LAMP1-related pathology, and the other vectors failing to provide a significant correction (Fig. 4, heart vessels inserts; Fig. 6G, O).

#### Cartilage and surrounding tissues

Alcian Blue staining of the knee joint did not reveal obvious pathology, although we observed a tendency toward an increased thickness of the distal GP of the femur of *Ids^y/−^* mice compared with WT mice (Fig. 5, Alcian Blue in GP; Fig. 6D; Supplementary Figs. S4 and S5). Cartilage tended to be thicker also in trachea, with tracheal chondrocytes showing disorganization and a more hypertrophic state in *Ids^y/−^* and *GFP*-treated mice compared with WT (Fig. 5, Alcian Blue in trachea; Fig. 6E; Supplementary Fig. S6). Alcian Blue staining was also increased in the tracheal mucosa (Mc) and adventitia (Ad), as well as in tissues surrounding the trachea such as IC of the thyroid gland (Th) and in the esophagus (Ep) in the submucosa (Sm) and in IC of the muscularis propria (Mp) (Fig. 5, Alcian Blue in trachea; Supplementary Fig. S6). Knee and trachea of *Ids^y/−^* mice also showed increased LAMP1 immunoreactivity (Fig. 5, LAMP1 in GP, AC, and trachea; Fig. 6K, L, M; Supplementary Figs. S7 and S8).

In the knee joint, LAMP1 immunoreactivity was detected in chondrocytes of the GP and the AC at levels that were 15- and 35-fold higher than in WT mice, respectively (Fig. 5, LAMP1 in GP and AC; Fig. 6K, L; Supplementary Fig. S6). We also observed increased LAMP1 staining in cells embedded in the TB and cortical bone (Fig. 5, GP inserts; Supplementary Fig. S7, small inserts), but not in the marrow (Fig. 5, LAMP1 in GP and AC; Supplementary Fig. S7). In trachea, we observed an upregulation of the LAMP1 signal in the chondrocytes of *Ids^y/−^* and *GFP*-treated mice at levels that were 10-fold higher than in WT mice (Fig. 5, LAMP1 in trachea; Fig. 6M; and Supplementary Fig. S8), in addition to a sparse upregulation of the LAMP1 immunoreactivity in the tissues surrounding the trachea such as the submucosa (Sm) and the muscularis propria (Mp) of the esophagus (Supplementary Fig. S8).

Gene therapy with all the vectors completely normalized pathology in cortical and trabecular bone, as well as in the tissues surrounding the trachea such as the esophagus and the thyroid, but had a limited effect on GP, AC, and tracheal cartilage pathology (Fig. 5, Fig. 6D, E, K, L, M; and Supplementary Figs. S4–S8). GPs of gene therapy-treated mice remained slightly thicker compared with WT animals (Fig. 6D), while tracheal cartilage thickness tended toward a reduction after gene therapy with *IDSco*, *IDS.IGF2co*, *IDS.ApoE2co*, although not to WT levels (Fig. 6E). Gene therapy had no effect on LAMP1 immunoreactivity levels in GP and tracheal cartilage (Fig. 6K, M). In the AC, we observed a threefold reduction of LAMP1 signal in gene therapy-treated mice compared with *GFP*-treated and untreated *Ids^y/−^* animals, although at levels ∼10-fold higher than those detected in WT mice (Fig. 6L).

These data show that the ability of lentiviral gene therapy to correct most of the peripheral pathology of *Ids^y/−^* mice, although correction of cartilage tissues was either partial (AC) or absent (GP and trachea) with all the vectors tested. Importantly, pathology of the great vessels of the heart could be effectively treated only by the *IDS.IGF2co* vector (complete correction) and the *IDS.ApoE2co* vector (near complete correction).

## DISCUSSION

In this study, we examined the correction of peripheral pathology after HSPC-LVGT and tracked the distribution of both tagged and untagged IDS proteins. Below, we discuss the treatment of peripheral pathology that has the largest impact on patient survival and quality of life.

Valvular heart disease affects 50–60% of the MPS II population and is one of the least responsive to ERT, with HV failure being the second most common cause of death for patients.^1,27^ Preclinical ERT tests on HV pathology in mice showed no correction of glycosaminoglycan (GAG) accumulation in other MPSs, while results for MPS II have not been reported.^28–31^ This suggests that delivery of recombinant lysosomal enzymes to HV when administrated intravenously is inefficient.^32^ In this study, we showed that HSPC-LVGT effectively treats Alcian Blue reactivity as well as lysosomal pathology of HV, as previously shown,^33^ independently of the tag used. IDS immunoreactivity was detected as strong signal in the endothelial layer of the HV leaflet, while the rest of the valve tissue was devoid of IDS protein. We hypothesize that these cells represent donor-derived IDS-expressing cells in the endothelium of the HV, which should be confirmed in future work.

In line with this hypothesis, hematopoietic stem cell-derived cells have been reported in the HV both in healthy and disease conditions,^34–36^ and recent reports assessing the effects of allogeneic BMT in MPS II patients also showed stabilization and a reduction in the frequency of valvular heart disease.^9,37,38^ We also detected Alcian Blue and LAMP1 reactivity in the TM of the great heart vessels such as the aorta, in agreement with the aorta narrowing and the systemic hypertension reported in patients with MPS II.^39–41^ Interestingly, the *IDS.IGF2co* vector was the only vector that completely corrected this pathology, with *IDS.ApoE2co* providing a near complete correction. Future work is required to elucidate the underlying mechanism of this phenomenon, but it is likely that IGF2-mediated increased uptake into smooth muscle cells of the TM through high-affinity binding to the CI-M6P/IGF2R, or a similar mechanism mediate by the ApoE2 tag and its cognate receptors, might play a role (as previously shown in MPS II fibroblasts and bEND.3 cells^15^).

Airway abnormalities are the main cause of death for MPS II patients and result from GAG accumulation in the upper respiratory airways, esophagus, mediastinal GAG storage, and thoracic skeletal abnormalities.^10,11,37,42,43^ GAG deposition in mucosa is among the causes of respiratory airway infection and malacia, and might also play a role in the onset of sleeping apnea.^11,42^

In this study, we showed complete correction of Alcian Blue and LAMP1 reactivity in the soft tissues surrounding the trachea, including the mucosa and the esophagus, after HSPC-LVGT with all the vectors tested. Consistently, we detected strong IDS staining in isolated cells in these tissues, suggesting engraftment of donor-derived cells, as discussed above. Allogeneic BMT was previously shown to improve upper and lower respiratory function, as well as obstructive apnea in MPS II patients, resulting in a reduction of airway infection from 83% to 50%, and reduction of sleep apnea from 63% to 11% in MPS II patients, but it was not successful in treating the central nervous system disease.^37^ This points to HSPC-LVGT as an advantageous therapy for preventing airway pathology.

However, tracheobronchomalacia is also caused by cartilage pathology,^10,11,37,42,43^ which, in our experiments, was not treated by HSPC-LVGT with any of the vectors tested. This is likely attributable to the limited ability of IDS protein to diffuse in cartilage, or to the ineffectiveness of HSPC-LVGT to achieve sufficient IDS protein concentrations at the cartilage interface to allow efficient diffusion. As correction of chondrocyte pathology relies on protein diffusion through the complex cartilage matrix, and diffusion is negatively affected by the cartilage thickness, the treatment of human cartilage by this approach will be even more challenging compared with mice considering the different thickness of cartilage in these two species (tracheal and AC thickness in humans is ∼100 times thicker compared with mice).^44^

Altogether, both HPSC-LVGT expressing tagged and untagged IDS proved capable of complete normalization of peripheral pathology in most tissues examined. Exceptions were HV, which were unresponsive, except when using *IDS.IGF2co* and *IDS.ApoE2co* as transgenes, and cartilage, which was largely unresponsive to any transgene tested.

## Supplementary Material

Supplemental data

## Data Availability

Data are available on request.
