# Supplementary material for: Lentiviral Gene Therapy for Mucopolysaccharidosis II with Tagged Iduronate 2-Sulfatase Prevents Life-Threatening Pathology in Peripheral Tissues But Fails to Correct Cartilage
Source: Hum Gene Ther. 2024 Apr 16;35(7-8):256–68. doi: 10.1089/hum.2023.177 (PMC11044872; doi:10.1089/hum.2023.177)
Supplement: Supplemental data [file Suppl_Data.pdf]

Materials and methods

Animals and Procedures

Animal experiments were performed as explained previously.[15] Briefly, heterozygous female B6N.Cg-Ids^{tm1Muen}/J mice were bred with wild-type C57BL/6J males to generate male hemizygous *Ids*$^{y/-}$ B6N.Cg-Ids^{tm1Muen}/J mice (hereafter *Ids*$^{y/-}$) and male wild-type B6N.Cg-Ids^{tm1Muen}/J littermates (hereafter WT). Heterozygous female *Ids*$^{+/-}$ CD45.1 were bred with wild-type male B6.SJL-*Ptprc*^{a} *Pepc*^{b}/BoyJ to generate male hemizygous *Ids*$^{y/-}$ B6N.Cg-Ids^{tm1Muen}/J mice (hereafter *Ids*$^{y/-}$ CD45.1) which were used as donor mice to distinguish donor and recipient cells via the *Ptprc*^{a} pan leukocytes marker, as previously described.45 Mice were bred according to standard procedures of the Laboratory Animal Science Center (EDC) at the Erasmus MC.[15] At the end of the experiment, mice were anesthetized and sacrificed by intracardiac perfusion with 50 ml of phosphate buffered saline (PBS). Relevant tissues were harvested and processed according to the follow-up analysis. All animal experiments in this study were approved by the Animal Experiments Committee (DEC) in the Netherlands and these complied with the Dutch legislature to use animals for scientific procedures.

Lentiviral vector construction and production

Vector construction was performed as described previously.[15] Briefly, codon-optimized human *IDS* and *IDS.ApoE2* (kindly provided by Prof. Dr. Brian Bigger) were cloned into pCCL-MND-c.o.RAG1[46] (kindly provided by Prof. Dr. Frank Staal) to generate pCCL-MND- *IDSco* (hereafter *IDSco*) and pCCL-MND- *IDS.ApoE2co* (hereafter *IDS.ApoE2co*) (in which RAG1 was removed). Codon optimized cassettes encoding IDS AA 530-550 fused to either Insulin-like growth factor 2 (IGF2; ALC GGE LVD TLQ FVC GDR GFY FSR PAS RVS RRS RGI VEE CCF RSC DLA LLE TYC ATP AKS E)[16,17] or to a minimal peptide of the human receptor associated protein (RAP12x2; AKI EKH NHY QK G AKI EKH NHY QK)[17] were subcloned

into the *IDSco* vector to generate pCCL-MND- *IDS.IGF2co* (hereafter *IDS.IGF2co*) and pCCL-MND- *IDS.RAP12x2co* (hereafter *IDS.RAP12x2co*). *GFP* sequence from the pRRL.PPT.SF.GFP.bPRE4*.SIN[16] vector was cloned into the pCCL-MND-c.o.RAG1.[46] Lentiviral particles were generated as previously shown.[47] Briefly, virus was produced by transfection of HEK29T cells and concentrated by ultracentrifugation (Beckman, SW32Ti rotor) of the medium supernatant at 20,000 rpm for 2 hours at 4°C. Functional viral titers were measured by transduction of HeLA cells as previously shown.[16]

IDS enzyme activity

IDS enzyme activity was performed as described previously.[15] Briefly, liver, kidney and spleen samples were disrupted with metal beads (stainless steel beads 5 mm, Qiagen) in 1 ml of double distilled-water by TissueLyser II (Qiagen, Venlo, the Netherlands) at 50 Hz for 5 mins. 100 µl of disrupted brain samples were diluted with 200 µl of 0.4% Triton X-100 (Sigma) and further disrupted with a Disruptor Genie (Scientific Industries) for 2 mins. Debris were pelleted at 12 000 g for 10 mins at 4°C. Supernatant was diluted 27 (liver and spleen) or 10 (kidney) times in water for measurement of IDS enzyme activity and 6 (liver and spleen) or 3 (kidney) times for measurement of total protein. Extracts from transfected HEK 293T cells and bone marrow were obtained in 100 µl of water by snap-freezing on dry-ice and mechanical disruption using pipetting. Debris was pelleted by centrifugation at 10,000 rpm for 5 minutes. Medium from HEK 293T cell transfection was centrifuged at 10,000 rpm for 5 minutes to remove debris. To obtain plasma, blood samples were mixed 3:1 with 4% Citrate Buffer (6132-04-3 Sigma) and plasma was separated by centrifugation at 2000 g for 10 minutes at 4 °C. Lysate from bone marrow was diluted 51 times in 0.2 % BSA for measurement of IDS enzyme activity and 6 times in water for measurement of total protein. Plasma was diluted 21 times in 0.2 % BSA for measurement of IDS enzyme activity and 31 times in water for measurement of total protein.

IDS activity was measured by incubation of 5 µl samples with 5 µl of 4-Methylumbelliferyl-α-L-idopyranosiduronic acid 2-sulphate disodium salt (Biosinth, Carbosynth; 2.5 mM in 0.2 M Na-acetate buffer, pH 5) and 5 µl of recombinant human α-L-Iduronidase (5 µg/ml in 0.1% BSA; R&D Systems) for 4 hours at 37 °C.[48] During each IDS activity measurement, dilutions of Elaprase at 1, 0.1, 0.01, 0.003 ng/µl were measured as a control for the linear range of detection of the assay.

Lentiviral hematopoietic stem cell transduction and transplantation procedures

HSPC isolation, transduction and transplantation was performed as described previously.[15] Briefly, bone marrow cells were harvested from 8-week to 4-month-old male *Ids*$^{y/-}$ CD45.1 mice. Hematopoietic stem and progenitor cells were enriched by lineage depletion using the Lineage Cell Depletion Kit – mouse (Miltenyi Biotec) and seeded at a density of 10^6 cells/ml in StemSpan SF expansion medium (Stemcell Technologies) supplemented with recombinant murine thrombopoietin (10 ng/ml, R&D systems), recombinant murine stem cell factor (100 ng/ml, R&D systems) and recombinant murine FMS-like tyrosine kinase 3 murine ligand (50 ng/ml, R&D systems). Cells were transduced over 24 hours at multiplicity of infection (MOI) of 1 with concentrated lentiviral particles and incubated at 37°C with 5 % CO_2. The day after, $1x10^6$ transduced Lin^- cells (200 µl of cells suspension in PBS) were transplanted intravenously into 8/11 weeks-old male *Ids*$^{y/-}$ CD45.2 recipients, previously subjected to 9 Gy total body irradiation (TBI) using the Gammacell 40 irradiator (Atomic Energy of Canada LTD, Ontario, Canada). No normalization for body weight was applied to the number of cells transplanted.

Histopathology and immunohistochemistry

After perfusion, liver, spleen, kidney, trachea and heart were excised and fixed in methacarn (v/v – 60 % absolute methanol, 30 % chloroform, 10% glacial acetic acid), dehydrated in 50%

and 70 % ethanol for 24 h and processed in paraffin (histokinette). Heart was cut along the long plane axis and both halves were embedded with the medial and later sides upwards. The knee joint was dissected intact and freed from the surrounding muscles. The knee joint was fixed in 4 % paraformaldehyde in PBS, washed in PBS, decalcified in 0.5 M EDTA (Sigma) pH 7.4 for 14 days, dehydrated in 50% and 70 % ethanol for 24 h and processed in paraffin (histokinette). Sectioning protocols: (1) liver, kidney and spleen were cut at a thickness 10 µm. Briefly, 5 serial sections were taken as soon as the tissues was cut, following by discarding of 5 serial 30 µm sections. 5 serial sections were taken afterwards. This process was repeated until completion of the tissue. (2) heart was cut at 8 µm-thickness. Briefly, 10 serial 8 µm sections were cut, following by discarding of 4 serial 10 µm and 1 8 µm sections. 10 serial 8 µm sections were taken afterwards. This process was repeated until completion of the tissue. (3) bones and trachea were cut at 10 µm until completion of the tissue and without discarding any section. Tissues were either stained with alcian blue, Safranin O/Fast green, or processed for immunohistological staining. For alcian blue staining, sections were rehydrated and equilibrated in 0.1 N hydrochloric acid (Sigma) for 30 s, followed by staining in 1 % Alcian Blue 8GX (Sigma) pH 1 for 30 mins. Sections were incubated in 0.1 N hydrochloric acid (Sigma) for 30 s and stained in 0.1 % nuclear fast red (Sigma) in 0.06 M aluminum sulfate hexadecahydrate (Sigma) for 5 mins. Sections were rinsed in 95 % ethanol, dehydrated and mounted in Entellan mounting medium (Sigma). Alcian blue pathology in brain was scored based on the scoring rules described in Tables S1, S2 and S3 by two independent operators blinded to the experimental and control groups. Safranin O/Fast green staining was performed by incubation in hematoxylin for 5 mins, removal of excess staining in distilled water, and incubation in 1 % acid-alcohol (1 % HCl in 70 % EtOH). After washing, sections were stained in 0.02 % Fast green (Sigma) for 1 min, incubated in 1 % acetic acid in 70 % EtOH and stained in 1 % Safranin O (Sigma) for 10 min. Sections were rinsed in 95 % ethanol, dehydrated and

mounted in Entellan mounting medium (Sigma). For immunohistochemical staining, sections were rehydrated and blocked for endogenous peroxidase in 3 % hydrogen peroxidase (dilution 1:2 in dH20 of a 6 % v/v solution BMS-2110-1E, PHC Corporation). Endogenous avidin and biotin were blocked for 15 mins at room temperature according to the manufacturer's instructions using an Avidin/Biotin blocking kit (SP-2001, Vector Laboratories), followed by blocking for 30 mins in staining buffer (3 % BSA, 3 % goat serum, 0.3 % Triton X-100 in PBS) at room temperature. Sections were stained with primary antibodies detecting either LAMP1 (rat anti-LAMP1,1:500, ab25245 Abcam), CD68 (rat anti-CD68 IgG, 1:300, MCA1957T BioRad), IDS (goat anti-human IDS biotinylated, 0.5 μg/ml, BAF2449, R&D systems) in staining buffer O/N at 4°C. The day after, sections were incubated with goat anti-rat antibody biotinylated (to detect CD68 and LAMP1, 1:200, 554014 BD Pharmingen). Sections were then incubated with streptavidin-HRP (1:50, DY998 R&D systems) in staining buffer for 60 mins at room temperature. Sections were finally incubated in impact DAB (SK-4105) for 2 mins (CD68, LAMP1, IDS all the organs) or 30 seconds (LAMP1 in trachea). Sections were mounted in Entellan (Sigma) and scanned by a NanoZoomer 2.0 (Hamamatsu Photonics, Japan). In growth plate, articular cartilage and trachea, LAMP1 intensity was measured using ImageJ. LAMP1 and CD68 were scored in liver and kidney (LAMP1 and CD68), as well as spleen (LAMP1) using the scoring rules shown in table S4 and S5 (LAMP1) and S6 (CD68) by two independent operators blinded to the experimental and control groups.

Flow cytometry analysis of chimerism

Flow cytometry analysis of chimerism in bone marrow was performed as described previously[15] using FITC-mouse anti mouse CD45.2 (BD bioscience, 553772) and PE-mouse anti-mouse CD45.1 (BD bioscience, 553776). Every experiment was performed with single-staining controls, unstained controls and isotype-stained controls (mouse IgG2a-FITC, BD

bioscience 349051; mouse IgG2a-PE, 349053). Measurement of chimerism was performed using a BD LSRFortessa and a FACS DIVA software recording 20 000 or more events per sample, while analysis was performed using FlowJo v10.

Quantitative polymerase chain reaction of vector copy number and chimerism.

Vector copy number (VCN) in bone marrow and chimerism in liver were determined by quantitative polymerase chain reaction (qPCR) as shown previously.[15,49] Genomic DNA was extracted with the NucleoSpin Tissue kit (Macherey-Nagel, Düren, Germany) or by ethanol precipitation and 50 ng of genomic DNA were used during a qPCR reaction with iTaq Universal SYBR Green Supermix (Bio-Rad, Hercules, CA). VCN was determined using primers specific for *PSI* (FW: 5'-*CAGGACTCGGCTTGCTGAAG*; RV: 5'-*TCCCCCGCTTAATACTGACG*) and mouse *Albumin* (FW: 5'-*ACTTTGAGTGTAGCA GAGAGGAACC*; RV: 5'-*CTCTTCACTGACCTAAGCTACTCCC*) and using a standard curve with logarithmic dilutions (10^9 – 10^2 copies in the reaction volume) of pCCL-MND- *IDSco* and pGEM-mAlbumin. The pGEM-mAlbumin plasmid was generated as described previously.[15] Chimerism was determined using primers specific for the *Cd45.1* allele (FW: 5'-*CTGAGCCTGCATCTAAACCTGATC*; RV: 5'-*TCACCTTCATAAAAGCCTTGT AGCTC*) as described previously.[15] Annealing temperature for analysis of chimerism was 67.5 °C, which resulted in specific amplification of the *Cd45.1* allele and no-amplification of the *Cd45.2* allele. Standard for chimerism analysis was prepared by mixing CD45.1 genomes with CD45.2 genomes at different ratios.[15] Reactions were performed and measured in a CFX96 real-time PCR detection system and analyzed by CFX Manager 3.0 (Bio-Rad, Hercules, CA).

Statistics

Statistical analysis was performed GraphPad Prism (version 9.0.0. for Windows, San Diego, California USA, www.graphpad.com). All results are presented as mean ± SEM and each data point is shown. Normality tests were performed by Shapiro-Wilk Test. Multiple comparison analysis was performed by one-way ANOVA with Bonferroni's correction.

References

45. Gleitz, H.F., Liao, A.Y., Cook, J.R., Rowlston, S.F., Forte, G.M., D'Souza, Z., O'Leary, C., Holley, R.J., and Bigger, B.W. (2018). Brain-targeted stem cell gene therapy corrects mucopolysaccharidosis type II via multiple mechanisms. EMBO Mol Med *10*. 10.15252/EMMM.201708730.
46. Garcia-Perez, L., van Eggermond, M., van Roon, L., Vloemans, S.A., Cordes, M., Schambach, A., Rothe, M., Berghuis, D., Lagresle-Peyrou, C., Cavazzana, M., et al. (2020). Successful Preclinical Development of Gene Therapy for Recombinase-Activating Gene-1-Deficient SCID. Mol Ther Methods Clin Dev *17*, 666–682. 10.1016/J.OMTM.2020.03.016.
47. Liang, Q., Vlaar, E.C., Catalano, F., Pijnenburg, J.M., Stok, M., Helsdingen, Y. van, Vulto, A.G., Unger, W.W.J., Ploeg, A.T. van der, Pijnappel, W.W.M.P.W.M.P., et al. (2022). Lentiviral gene therapy prevents anti-human acid α-glucosidase antibody formation in murine Pompe disease. Mol Ther Methods Clin Dev *25*, 520–532. 10.1016/J.OMTM.2022.04.016.
48. Voznyi, Y. v., Keulemans, J.L.M., and van Diggelen, O.P. (2001). A fluorimetric enzyme assay for the diagnosis of MPS II (hunter disease). J Inherit Metab Dis *24*, 675–680. 10.1023/A:1012763026526/METRICS.
49. Bergsma, A.J., Stijn, •, In 't Groen, L.M., Catalano, F., Yamanaka, M., Takahashi, S., Okumiya, T., Ans, •, Van Der Ploeg, T., and Pim Pijnappel, • W W M (2021). A generic assay for the identification of splicing variants that induce nonsense-mediated decay in Pompe disease. European Journal of Human Genetics *29*, 422–433. 10.1038/s41431-020-00751-3.

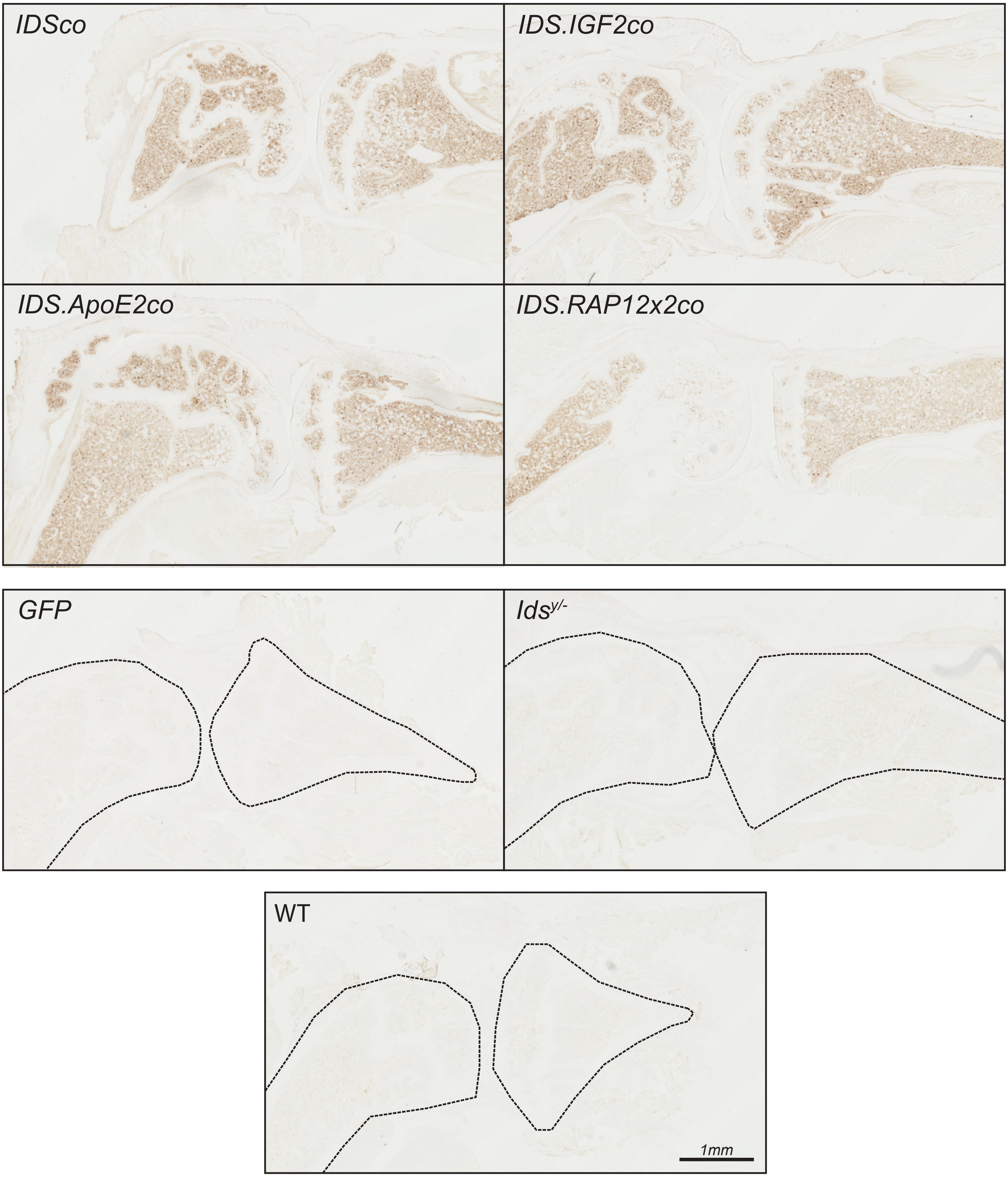

Figure S1. IDS staining of the knee joint.
Representative enlarged pictures of IDS staining in the articular knee cartilage of untreated and gene therapy treated mice and controls. Dashed lines indicate perimeter of bones in GFP, Idsy/- and WT. n=3. Scale bars = 1 mm. n=3

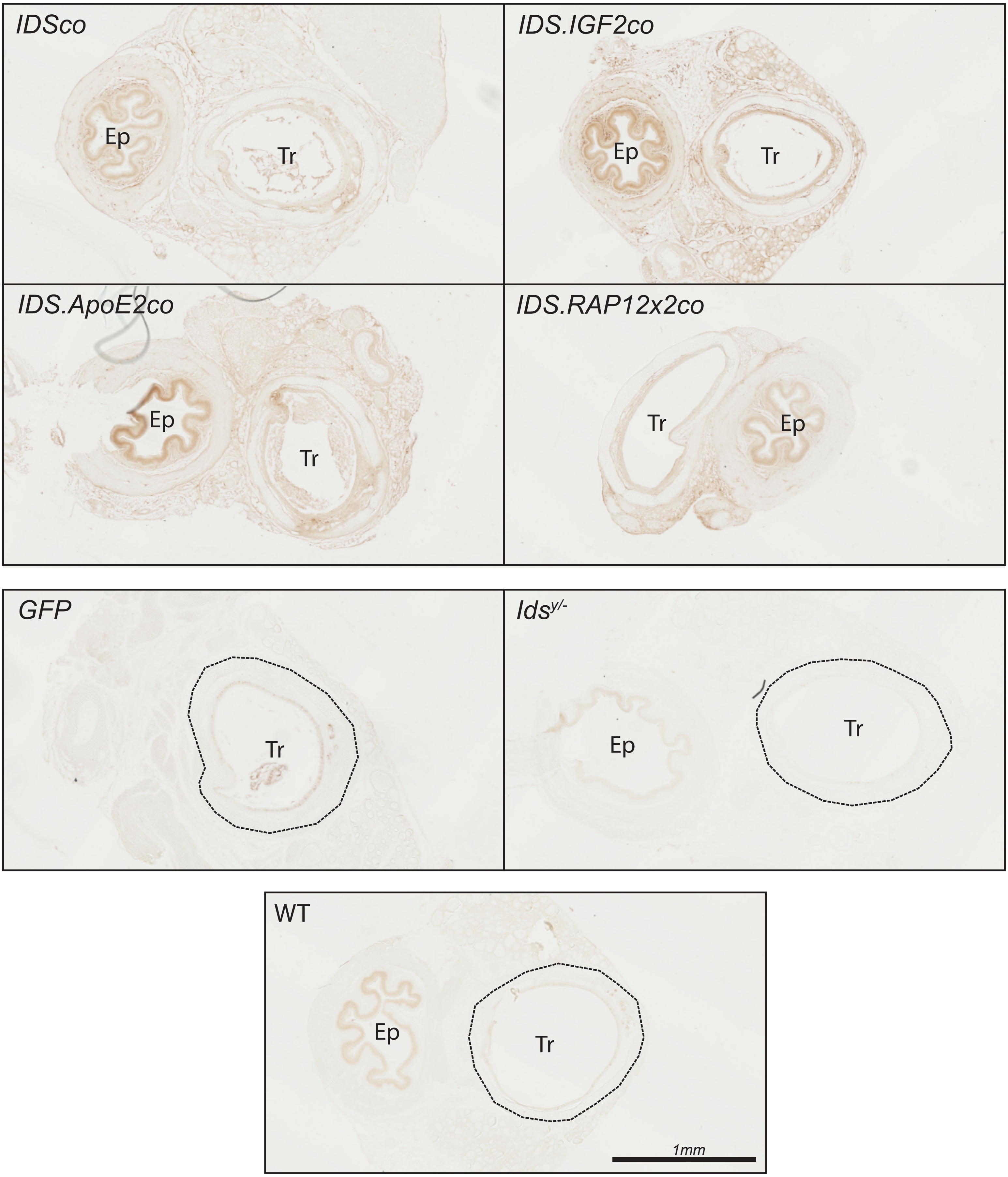

Figure S2. IDS staining of trachea.
Representative enlarged pictures of IDS staining in trachea of untreated and gene therapy treated mice and controls. Ep: esophagus; Tr: trachea. Dashed lines indicate the perimeter of the tracheal cartilage in GFP, Idsy/- and WT. n=3. Scale bars = 1 mm. n=3

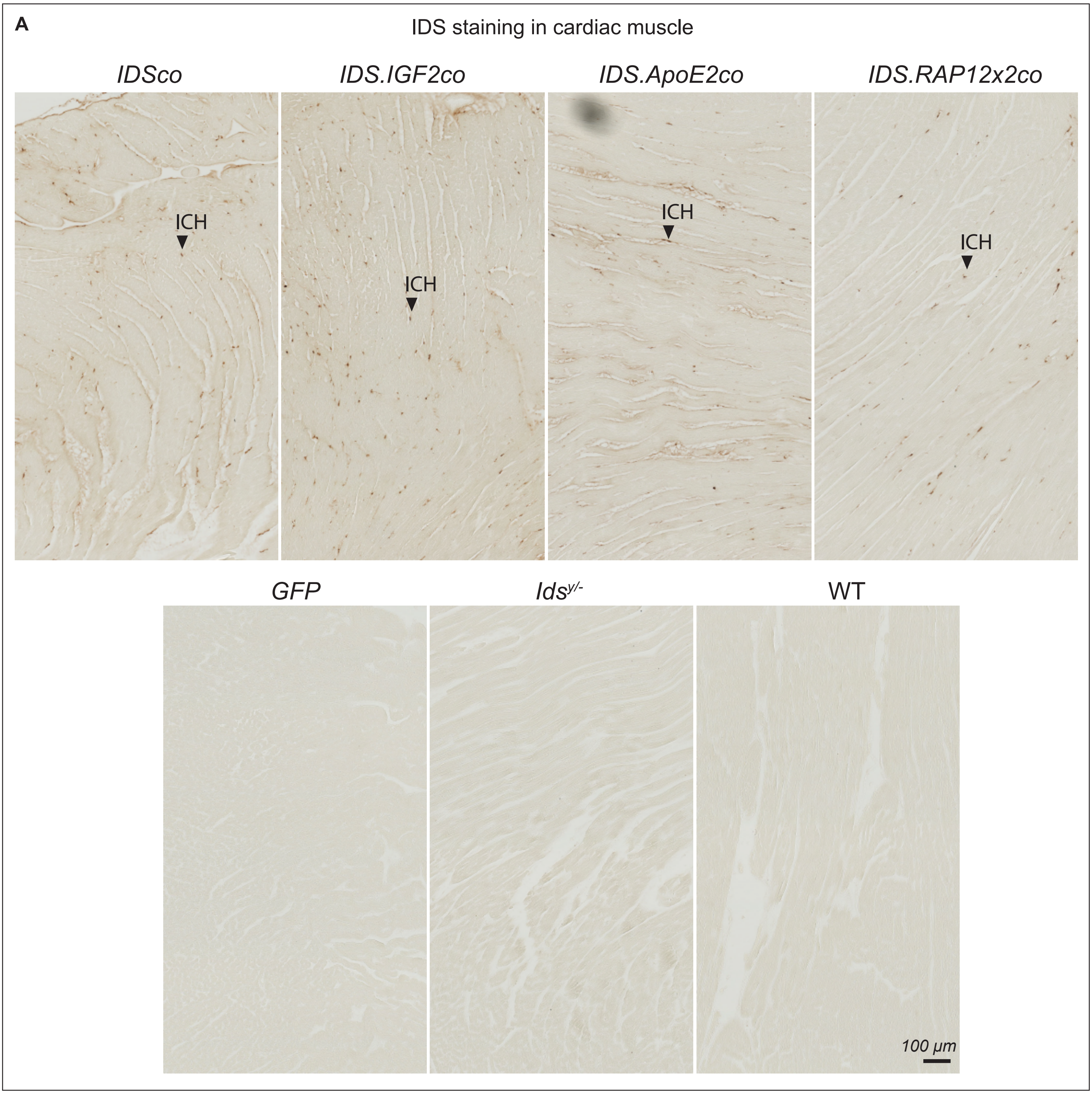

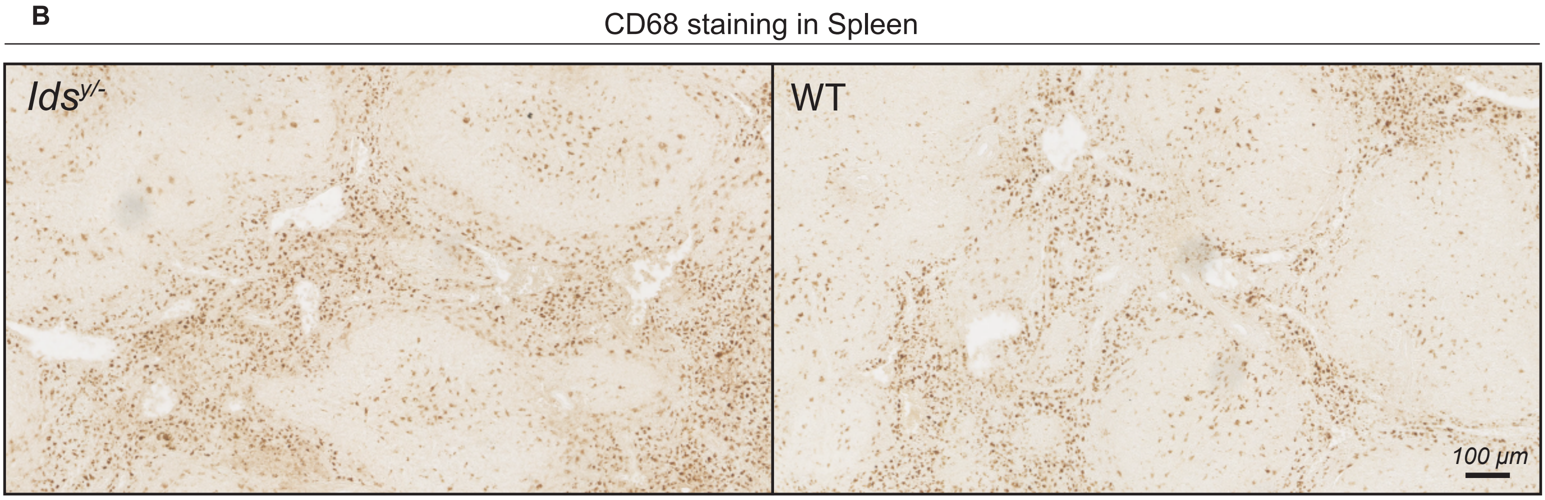

Figure S3. IDS staining in cardiac muscle and comparison of CD68 deposition in spleen of Idsy/- and WT mice.

(A) Representative pictures of IDS staining in cardiac muscle after gene therapy and controls and (B) CD68 staining in spleen of Idsy/- and WT mice. ICH: interstitial cell of the heart muscle. n=3. Scale bars = 100 µm. n=3

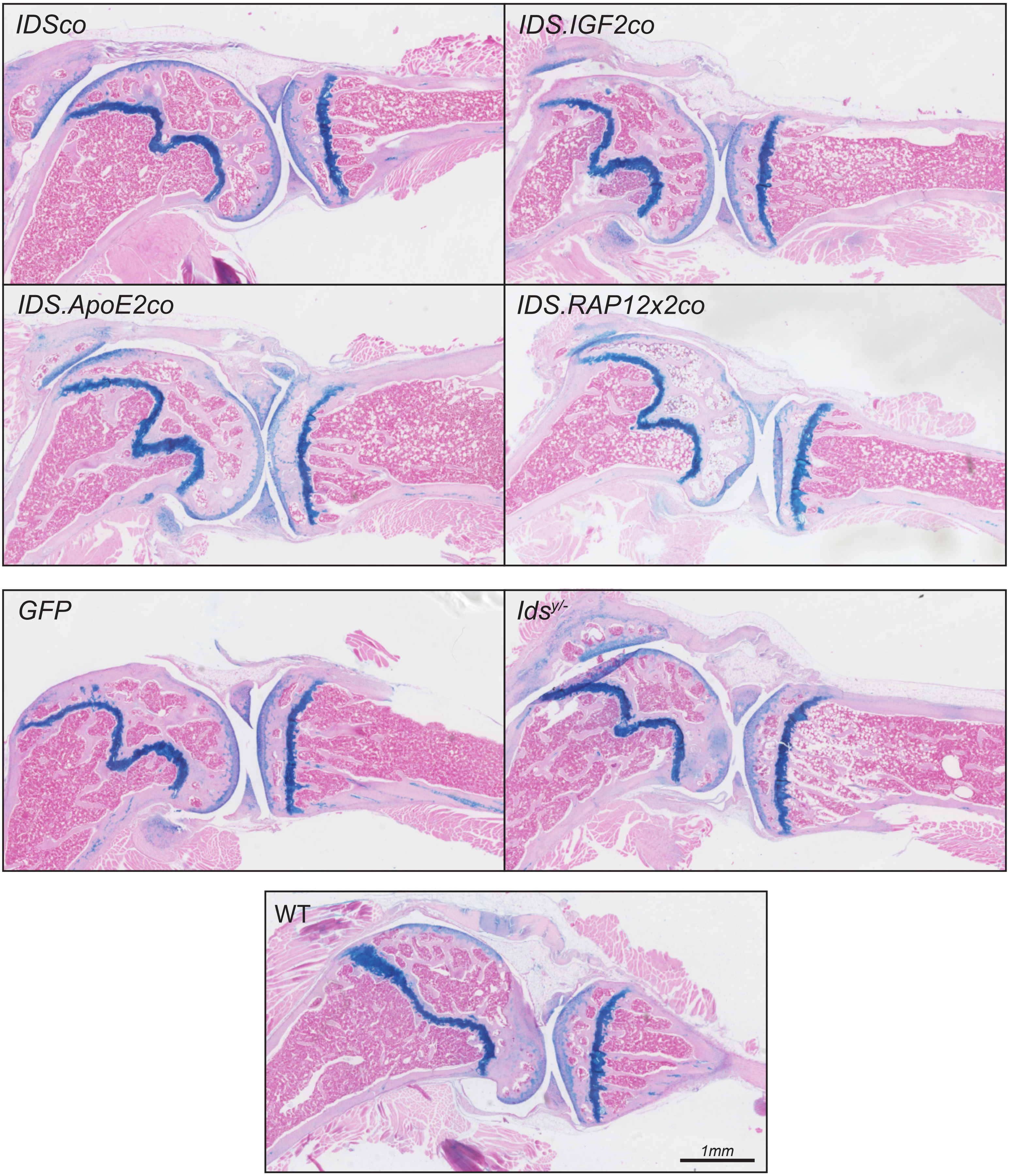

Figure S4. Alcian blue staining of the knee joint.
Representative enlarged pictures of articular knee cartilage stained with alcian blue of untreated and gene therapy treated mice and controls. n=3. Scale bars = 1 mm. n=3

Safranin O / Fast green staining of bones

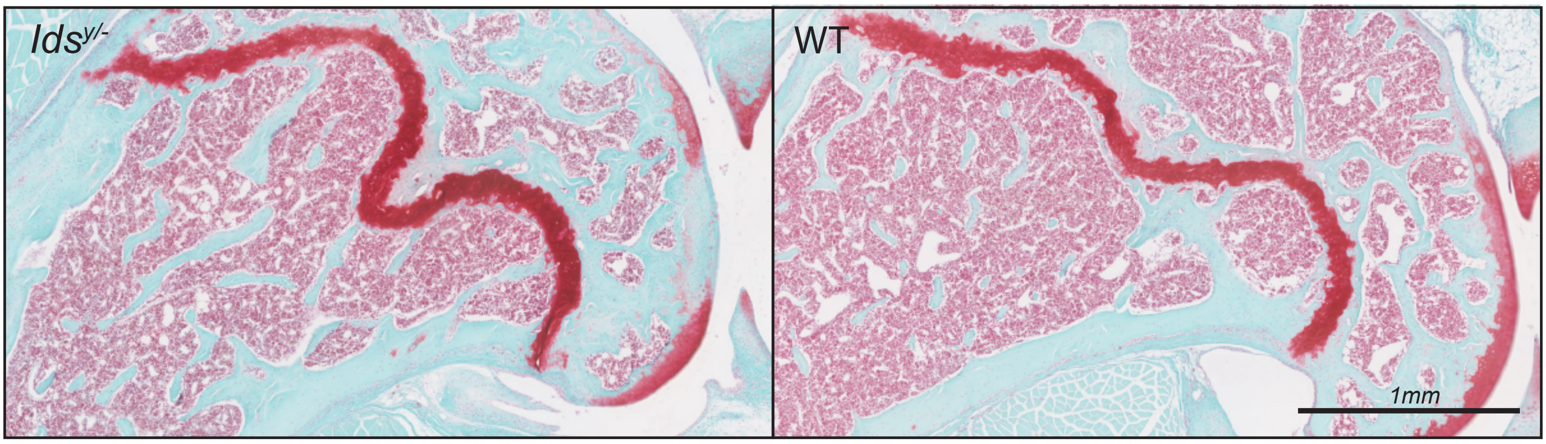

Figure S5. Comparison of Safranin O/Fast green staining in Idsy/- and WT tibia.
Representative enlarged pictures of the proximal epiphysis of the tibia of Idsy/- and WT mice stained with Safranin O/ Fast green. n=3. Scale bars = 1 mm. n=3

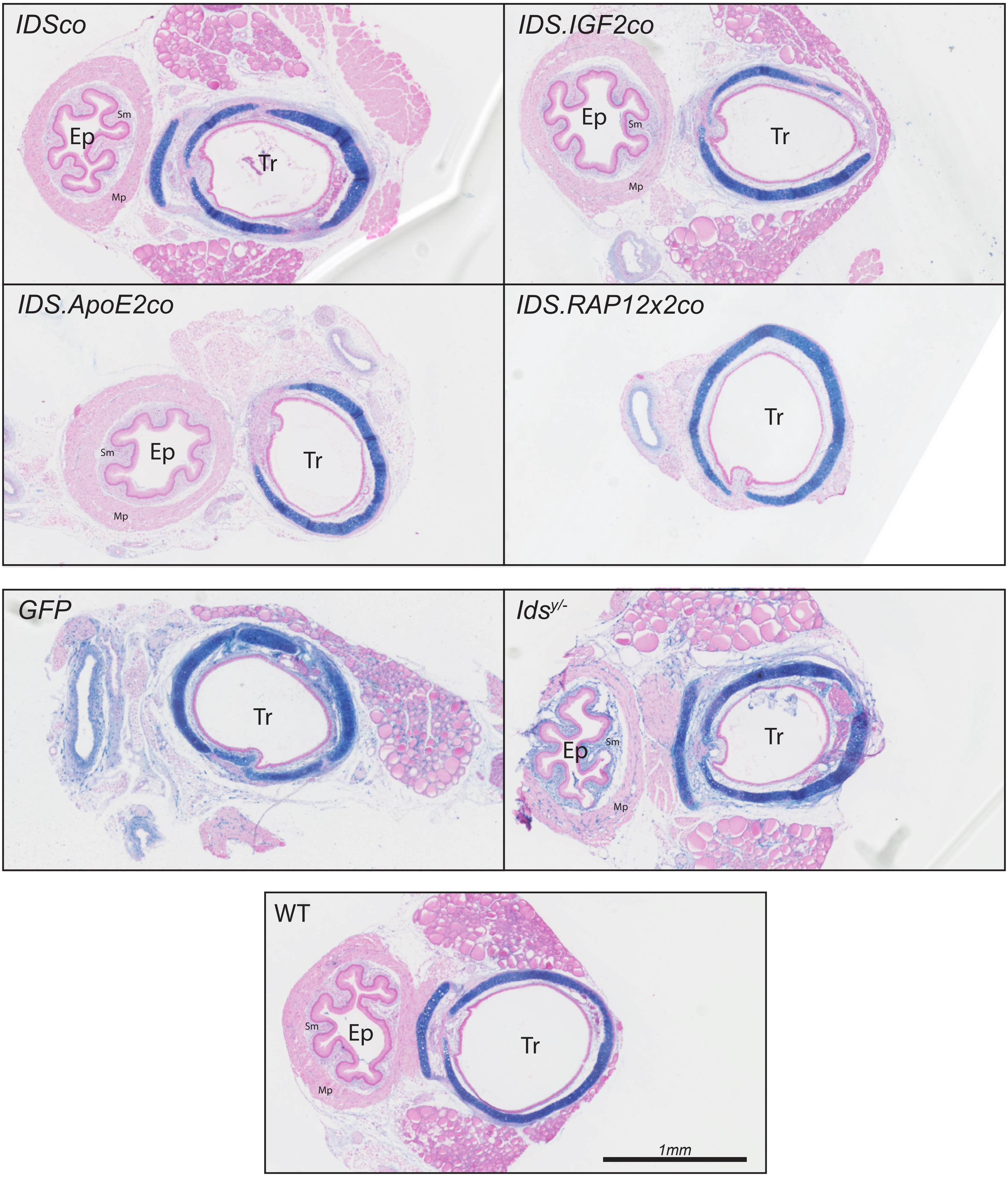

Figure S6. Alcian blue staining of trachea.
Representative enlarged pictures of alcian blue staining in trachea of untreated and gene therapy treated mice and controls. Ep: esophagus; Tr: trachea; Sm: esophageal submucosa; Mp: esophageal muscularis propria. n=3. Scale bars = 1 mm. n=3

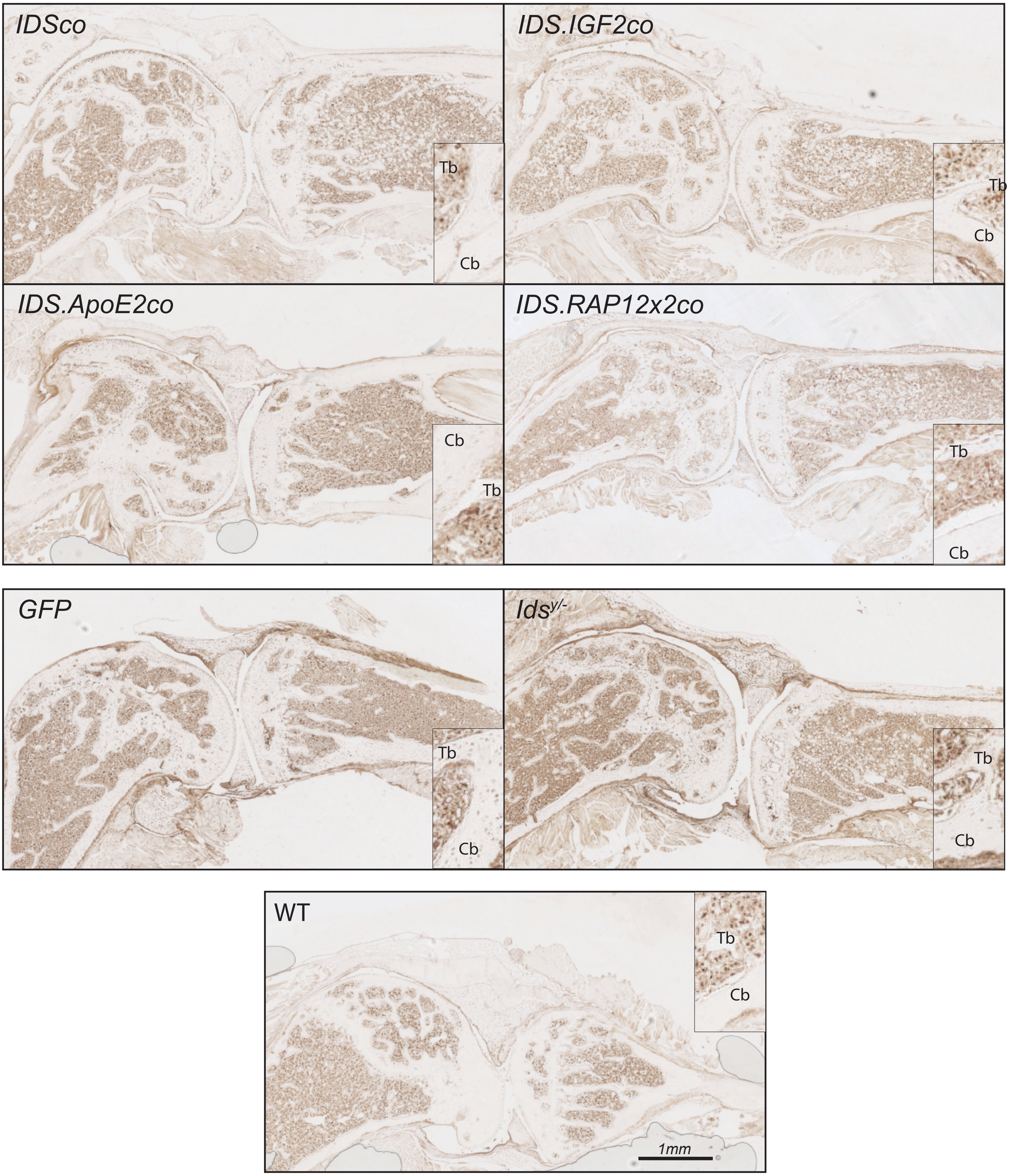

Figure S7. LAMP1 staining of the knee joint.
Representative enlarged pictures of LAMP1 staining in the articular knee cartilage of untreated and gene therapy treated mice and controls. Tb: trabecular bone; Cb: cortical bone. Inserts represent regions of interest magnified 4 times. n=3. Scale bars = 1 mm. n=3

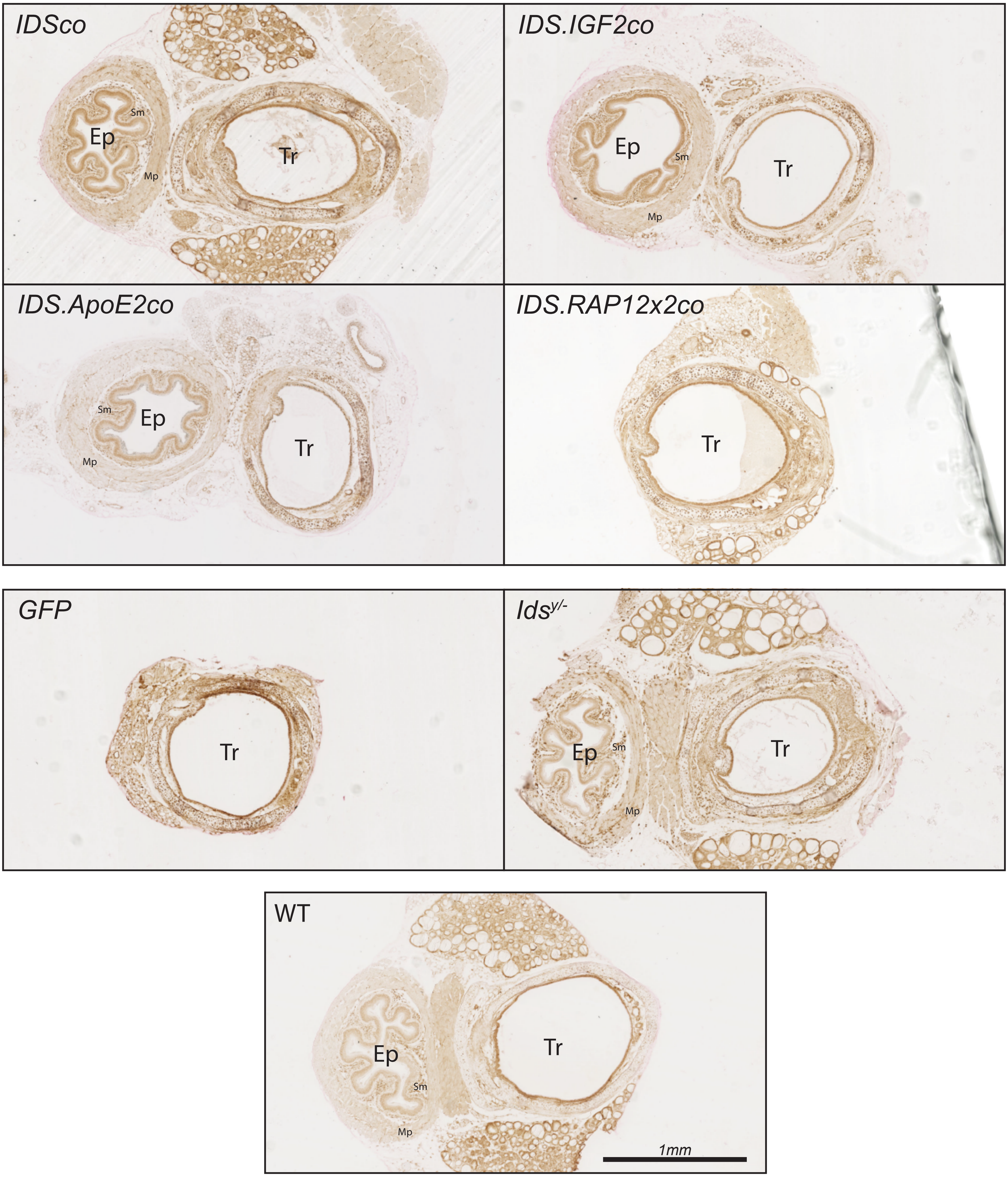

Figure S8. LAMP1 staining of trachea.
Representative enlarged pictures of LAMP1 staining in trachea of untreated and gene therapy treated mice and controls. Ep: esophagus; Tr: trachea. n=3. Scale bars = 1 mm. n=3

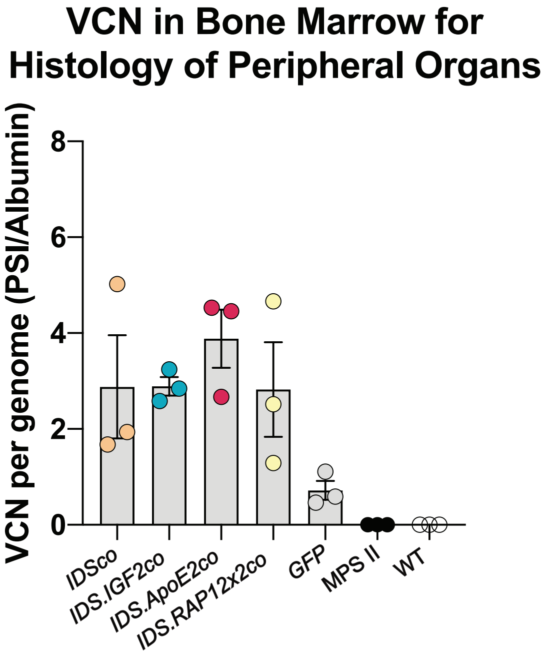

Figure S9. VCN in bone marrow of the mice used for histology of the peripheral organs.
VCN in bone marrow of gene therapy-treated Idsy/- mice and control mice used for histology of the peripheral organs (IDS, Alcian Blue, LAMP1, CD68, Safranin O/Fast Green). VCN per genome was measured by qPCR on PSI and Albumin loci. Data are presented as means ± SEM and were analyzed by one-way ANOVA with Bonferroni's correction. n=3.

Table S1	Scoring of alcian blue staining in Spleen, Liver and Kidney
Score	Rules
1	0 Alcian blue positive cells in 5 random fields
2	A total of 1-5 alcian blue positive cells in 5 random fields
3	A total of 5-10 alcian blue positive cells in 5 random fields
4	A total of 10-15 alcian blue positive cells in 5 random fields
5	A total of 15-30 alcian blue positive cells in 5 random fields
6	more than 100 Alcian blue positive cells in 1 random field

Table S2	Scoring of alcian blue staining in Heart Valves
Score	Rules
1	Weak alcian blue staining
2	Moderate alcian blue staining
3	Strong alcian blue staining

Table S3	Scoring of alcian blue staining in Great Heart Vessels
Score	Rules
1	No alcian blue staining
2	Alcian blue staining in less than 5 % of the aortic wall area
3	Alcian blue staining in less than 30 % of the aortic wall area
4	Alcian blue staining in less than 60 % of the aortic wall area
5	Strong alcian blue staining in all the aortic wall area

Table S4	Scoring scoring of Lamp1 in Liver and Spleen
Score	Rules
1	Weak Lamp1 staining in sinuisoidal cells
2	Moderate Lamp1 staining in sinuisoidal cells
3	Strong Lamp1 staining in sinuisoidal cells

Table S5	Scoring scoring of Lamp1 in Kidney
Score	Rules
1	Strong Lamp1 staining in the glomerular parietal sheet and no staining in the glomerular tuft; some tubules show Lamp1 staining
2	Strong Lamp1 staining in the glomerular parietal sheet and staining in some cells of the glomerular tuft; some tubules show Lamp1 staining
3	Moderate Lamp1 staining in the glomerular parietal sheet and widespread staining in the glomerular tuft; an increased number of tubules show Lamp1 staining

Table S6	Scoring scoring of CD68 in Liver and Kidney
Score	Rules
1	Weak Cd68 staining in some cells
2	Weak Cd68 staining in several cells
3	Strong Cd68 staining in several cells
